# Untargeted metabolomics using liquid chromatography-high resolution mass spectrometry and chemometrics for analysis of non-halal meats adulteration in beef meat

**DOI:** 10.5713/ab.23.0238

**Published:** 2024-01-14

**Authors:** Anjar Windarsih, Nor Kartini Abu Bakar, Abdul Rohman, Nancy Dewi Yuliana, Dachriyanus Dachriyanus

**Affiliations:** 1 Department of Chemistry, Faculty of Science, Universiti Malaya, Kuala Lumpur 50603, Malaysia; 2 Research Center for Food Technology and Processing (PRTPP), National Research and Innovation Agency (BRIN), Gunungkidul, Yogyakarta 55861, Indonesia; 3 Department of Pharmaceutical Chemistry, Faculty of Pharmacy, Universitas Gadjah Mada, Yogyakarta 55281, Indonesia; 4 Center of Excellence, Institute for Halal Industry and Systems (PUIPT-IHIS), Universitas Gadjah Mada, Yogyakarta 55281, Indonesia; 5 Department of Food Science and Technology, IPB University, Bogor 16680, Indonesia; 6 Faculty of Pharmacy, Andalas University, Padang 25175, Indonesia

**Keywords:** Beef Meat, Chemometrics, Halal Authentication, LC-HRMS, Untargeted Metabolomics

## Abstract

**Objective:**

The adulteration of raw beef (BMr) with dog meat (DMr) and pork (PMr) becomes a serious problem because it is associated with halal status, quality, and safety of meats. This research aimed to develop an effective authentication method to detect non-halal meats (dog meat and pork) in beef using metabolomics approach.

**Methods:**

Liquid chromatography-high resolution mass spectrometry (LC-HRMS) using untargeted approach combined with chemometrics was applied for analysis non-halal meats in BMr.

**Results:**

The untargeted metabolomics approach successfully identified various metabolites in BMr DMr, PMr, and their mixtures. The discrimination and classification between authentic BMr and those adulterated with DMr and PMr were successfully determined using partial least square-discriminant analysis (PLS-DA) with high accuracy. All BMr samples containing non-halal meats could be differentiated from authentic BMr. A number of discriminating metabolites with potential as biomarkers to discriminate BMr in the mixtures with DMr and PMr could be identified from the analysis of variable importance for projection value. Partial least square (PLS) and orthogonal PLS (OPLS) regression using discriminating metabolites showed high accuracy (R^2^>0.990) and high precision (both RMSEC and RMSEE <5%) in predicting the concentration of DMr and PMr present in beef indicating that the discriminating metabolites were good predictors. The developed untargeted LC-HRMS metabolomics and chemometrics successfully identified non-halal meats adulteration (DMr and PMr) in beef with high sensitivity up to 0.1% (w/w).

**Conclusion:**

A combination of LC-HRMS untargeted metabolomic and chemometrics promises to be an effective analytical technique for halal authenticity testing of meats. This method could be further standardized and proposed as a method for halal authentication of meats.

## INTRODUCTION

Meat adulteration using non-halal meats is a serious matter because certain religions such as Muslim and Judaism are not allowed to consume non-halal foods. Moreover, Muslim applies the strict regulation regarding halal products according to the Shariah law. Halal is not only associated with religion but also to a healthy life style [1]. The demand for halal foods has been reported to be increasing worldwide due to their quality and safety [2]. Beef (BMr) is one of the favorite meats consumed by people around the world due to its high nutrition and taste. In market place, the price of BMr is higher compared to other meats, thus make it prone for adulteration [3]. Non-halal meat such as dog meat (DMr) and pork (PMr), are widely spread in Southeast Asia countries including Indonesia, Malaysia, and Thailand. These meats have a lower price and similar physical appearances as beef. DMr and PMr are often mixed with beef and labelled as pure beef to get the additional profits by unethical players and producers [4]. This is a serious problem because it could harm consumers.

Various analytical methods such as gas chromatography [5], liquid chromatography [6], Fourier transform infrared spectroscopy [7], near infrared spectroscopy [8], Raman spectroscopy [9], and DNA-based methods [10] have been developed for the detection of non-halal meats in meats and meat-based products. Methods based on molecular analysis such as polymerase chain reaction (PCR) and real-time PCR to detect a DNA target have been widely applied for meat authentication purposes, some countries even apply RT-PCR as the standard method [11]. However, several factors such as processing treatments and temperature could degrade DNA, therefore, sometimes the non-halal DNA can not be detected using RT-PCR [12,13]. Moreover, RT-PCR technique is not a simple technique because of its complex sample preparation steps and costly reagents [14]. Therefore, the development of powerful and robust analytical methods to detect non-halal meats with high accuracy and high reproducibility is a must.

Recently, the omics technology such as metabolomics has been widely applied in numerous research including analysis and authentication of foods [15]. Metabolomics is a comprehensive study intended to identify an enormous number of metabolites from certain samples at a particular condition [16]. Metabolomics could be used for the identification of metabolites composition in meats and to investigate the difference of metabolites among meats [17]. The untargeted metabolomics approach demonstrates some advantages for authentication of food products due to its capability to screen as many metabolites as possible in food products. Liquid chromatography-high resolution mass spectrometry (LC-HRMS) has been used for untargeted metabolomics research because of its capability for metabolites screening with high throughput capacity. Moreover, it has high sensitivity, high specificity, and high resolving power for metabolomics analysis [18].

The advanced statistical tools such as chemometrics, is obviously required for untargeted metabolomics approach due to the vast amount of data resulting from an untargeted measurement. Chemometrics could be used to handle and process the huge data from untargeted LC-HRMS metabolomics analysis [19]. Some of the chemometrics techniques such as pattern recognition using principal component analysis (PCA), discriminant analysis (DA), partial least square discriminant analysis (PLS-DA), soft independent modelling class analogy (SIMCA) along with multivariate calibrations of partial least square (PLS) and principle component regression and many more [20] have been used during food authentication. The combination of metabolomics analysis using Liquid chromatography-mass spectrometry (LC-MS) based method and chemometrics has been widely used in various research for food authentication including halal authentication analysis of meats. Chemometrics analysis could be used to identify halal and non-halal samples based on their metabolite patterns. Moreover, chemometrics has the capability to investigate potential biomarkers which are very useful for the identification and differentiation of the studied samples [20,21].

Differentiation of chicken meats obtained from two slaughtering methods, zabiha (halal) and non-zabiha (non-halal) was successfully performed using untargeted LC-HRMS metabolomics [22]. A study on the metabolite differences between chicken meats obtained from normal slaughtering and dead-on chicken meats has also been performed by Sidwick et al [23] using LC-HRMS combined with chemometrics. Moreover, detection of pork adulteration in beef by focusing on lipid metabolites has been investigated by using LC-HRMS untargeted approach. Some potential biomarkers important for such differentiation also could be extracted using chemometrics analysis [24]. The presence of pork as adulterant in beef meatballs has also been successfully analyzed using LC-HRMS and pattern recognition chemometrics by employing an untargeted metabolomics approach. Moreover, multivariate regression was applied to create a prediction model [25]. However, studies on the analysis of non-halal meats such as pork and dog meat in high quality of halal meats using an untargeted LC-HRMS metabolomics and chemometrics are still limited.

To our best knowledge, there is no report on the analysis of DMr and PMr adulteration in beef for halal authentication using a non-targeted LC-HRMS metabolomics combined with chemometrics. Therefore, the main objective of this study was to develop an analytical method for halal authentication of beef from DMr and PMr adulteration by employing an untargeted LC-HRMS metabolomics. In addition, chemometrics was applied to identify discriminating metabolites potential for biomarkers important for the differentiation of samples.

## MATERIALS AND METHODS

### Materials

All LC-MS hypergrade solvents (methanol, water, acetonitrile), HPLC grade solvent of methanol, and formic acid were obtained from Merck (Darmstadt, Germany). The standard solution of positive and negative calibration solution (Pierce LTQ positive and negative) for mass spectrometry were purchased from Thermo Fisher Scientific (Rockford, IL, USA).

### Sample collection and preparation

Beef (n = 5) sample was obtained from five different markets in Yogyakarta and Central Java, Indonesia. Pork (n = 3) and dog meat (n = 3) were purchased from three different meat sellers in Yogyakarta, Indonesia. The loin part was used for metabolomics analysis. The meat samples were stored at −20°C prior used for extraction. Each type of meat was ground using a meat grinder to obtain ground meat, then stored to −20°C until used for analysis. Each meat obtained from all locations were mixed. The samples of pure beef, pork, and dog meat as well as the binary mixtures of beef-pork and beef-dog meat were prepared in triplicates.

### Metabolite extraction

The extraction of metabolites for metabolomics analysis was carried out based on the method by Wang et al [14] subjected to slight modifications. Pure BMr samples and those adulterated with DMr and PMr using various concentration levels (0.1%, 1%, 5%, 10%, 25%, and 50% w/w) were prepared. The ground meat was weighed, and the mixtures were prepared in a total weight of 5 g. Samples were placed into a Beaker glass and added with methanol (25 mL). Subsequently, samples were vortexed for 60 s, then ultrasonicated for 30 min at room temperature. The protein was precipitated by storing the samples at −20°C for 1 hour. The supernatant was collected by centrifugation for 10 min at 4°C at 5,000 rpm. The 1 mL of supernatant was pipetted and filtered using PTFE filter 0.22 μm. The filtered supernatant was placed into a clear 2 mL HPLC vial. Three replicates of each sample were prepared.

### Metabolomics analysis

Metabolomics was performed according to our previous study with slight modifications [25]. Separation of compounds was carried out using an ultra-high performance liquid chromatography (UHPLC Vanquish; Thermo Scientific, Rockford, IL, USA) equipped with a binary pump. Two mobile phases consisted of water containing 0.1% formic acid (A) and methanol containing 0.1% formic acid (B) were used for eluting sample with the flow rate of 0.30 mL/min. An Accucore C-18 column (100 mm×2.1 mm×2.6 μm) was used for separating metabolites in samples which was injected at 10 μL. The temperature of column was set at 40°C during the compound’s separation. Elution was carried out using gradient mode as follows; at first, the mobile phase was set at 95% A, then continue to for 10% A at 16 min. Then, maintained at 10% A until 30 min before setting back to 95% A (35 min). The detection of compounds was performed using a high-resolution mass spectrometer (Orbitrap Q-Exactive; Thermo Scientific, USA). The flow rate setting for sheath gas was 32 arbitrary unit (AU), followed by auxiliary gas of 8 AU and sweep gas of 4 AU, respectively. The setting for parameter of capillary temperature was 320°C followed by gas heater temperature of 30°C. The ionization was performed in both electro spray ionization (ESI) positive and ESI negative modes with MS1 resolution of 70,000 and MS2 resolution of 17,500. The screening of compounds was carried out at m/z range of 66.7 to 1,000 m/z.

### Data processing

The Compound Discoverer (Thermo Scientific, USA) software was used to identify metabolites composition in BMr, DMr, PMr, and their mixtures. The raw total ion chromatogram (TIC) both of sample and blank was used for analysis. Spectrum selection, retention time alignment, feature detection, and metabolite identification were performed. The Chemspider and MzCloud database were used for database matching. Analysis was performed using an untargeted workflow. Only metabolites having full match MS2 fragmentation and error mass between −5 and 5 ppm were selected. The metabolite dataset was converted into an excel file for chemometrics analysis.

### Chemometrics analysis X

The selected metabolites from metabolite identification analysis were used as variables for chemometrics. The data were subjected to unit variance scaling prior to chemometrics analysis. Both pattern recognition and multivariate calibration chemometrics were applied. Chemometrics analysis was conducted using a SIMCA 14.1 (Umetrics, Umea, Sweden) software and Metaboanalyst 5.0 platform. Principal component analysis was performed and observed using PCA score plot, PCA loading plot, R^2^, and Q^2^ values. The PLS-DA was further used and evaluated using R^2^X, R^2^Y, Q^2^ values, loading score and loading plot. Validation tests such as permutation test, predicted residual error sum of squares (PRESS) value, and receiver operating characteristics (ROC) test were used for PLS-DA. The identification of discriminating metabolites potential as metabolite markers was conducted using variable importance for projection (VIP) test by identifying the variables with VIP more than 1.0. The metabolites were then subjected to analysis of variance (ANOVA) analysis. Metabolites were with p-value <0.05 were considered as significant metabolites in line with the increase of pork and dog meat levels. Multivariate calibration of PLS as well as its orthogonal form (OPLS) was used to build prediction model. The models were evaluated using the PLS plot, R^2^, and the residual plot. Validation of PLS dan OPLS was performed using root mean square error of cross validation (RMSECV) and root mean square error of estimation (RMSEE).

## RESULTS AND DISCUSSION

### Metabolomics analysis using LC-HRMS

**Figure 1:**
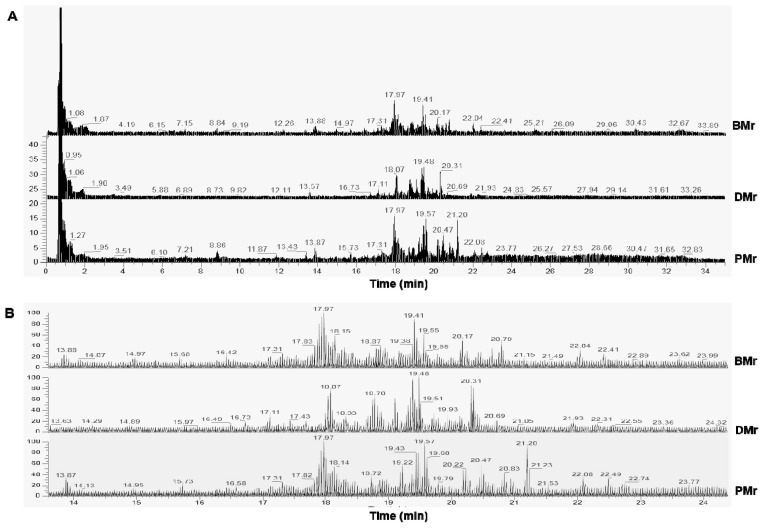
Total ion chromatogram (TIC) of beef meat (BMr), dog meat (DMr), and pork meat (PMr) measured at retention time between 0.0 and 35.0 min (A) and at retention time between 14.0 and 24.0 min (B).

**Figure 2:**
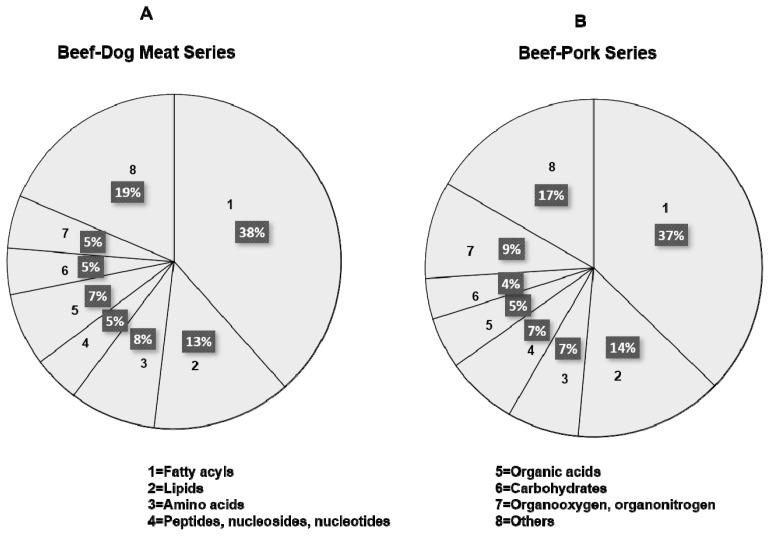
Classification and proportion of the identified metabolites in beef-dog meat series (A) and beef-pork series (B).

Metabolomics analysis using LC-HRMS employing an untargeted workflow revealed a wide number of metabolites contained in BMr, DMr, and PMr. Figure 1 shows the TIC from BMr, DMr, and PMr exported from XCalibur software. In general, the samples showed a similar TIC pattern, however, deep investigation found some peak differences at the retention time between 14.0 and 24.0 min. Analysis on the TIC against the databases using Compound Discoverer software could retrieve the metabolite compositions on each sample. Figure 2 illustrates the identified metabolites from beef-dog meat and beef-pork series measured using LC-HRMS metabolomics. Fatty acyls class placed at the highest proportion followed by lipids in both sample series which consist of various types of fatty acids and polar lipids such as phospholipids. Various types of amino acids and organic acids were also identified. The results was in accordance with previous reports as fatty acids, lipids, and amino acids were reported to be present in high amount in meats [26]. In addition, it was found that creatine had the highest peak area in all types of meat (BMr, DMr, and PMr), followed by carnitine. It was in agreement with previous research reporting that meats such as beef, pork, and chicken contained high amount of creatine and carnitine [27]. Due to the complexity of the identified metabolites in BMr, DMr, and PMr samples, therefore, an advanced statistical tool is required to analyze the data for the authentication purposes to guarantee the results.

### Chemometrics of pattern recognition

**Figure 3:**
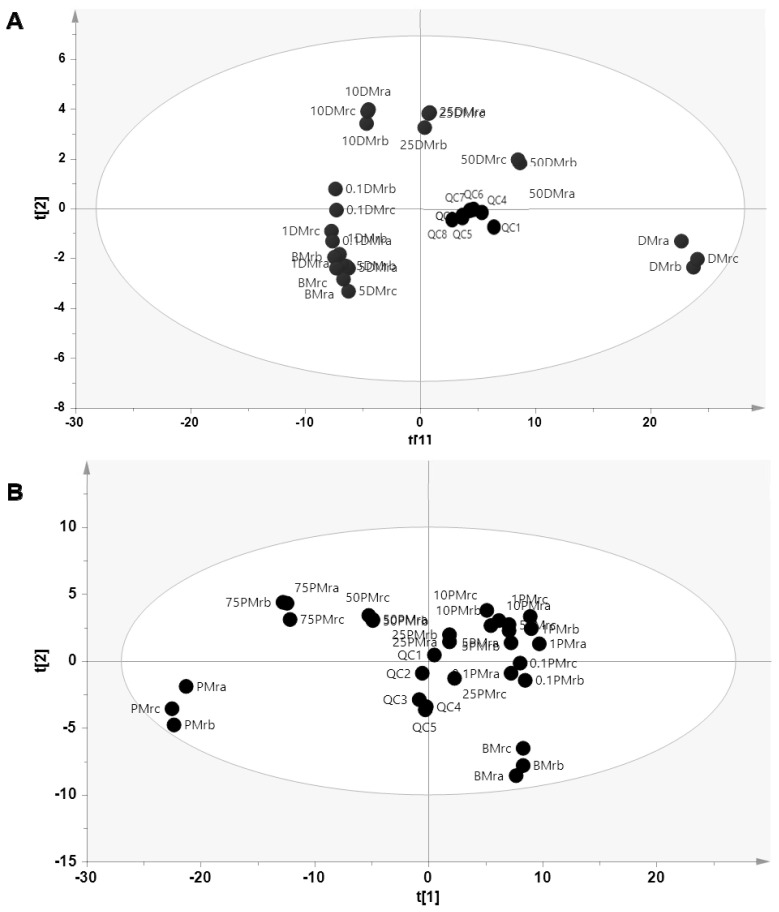
The score plot of principal component analysis to differentiate authentic beef from dog meat adulteration (A) and pork adulteration (B). BMr = 100% beef; DMr = 100% dog meat; PMr = 100% pork, the number before samples code indicates the percentage of adulterant added in beef; QC = quality control samples.

In this study, PCA was first applied to observe the pattern of samples grouping based on their metabolite compositions. Using seven principal components (PCs), PCA could differentiate pure BMr samples with those adulterated with DMr at several concentration levels, especially in high levels of DMr (10%, 25%, and 50%) with R^2^ = 0.877 and Q^2^ = 0.577. However, in the presence of low concentration of DMr (0.1%, 1%, and 5%), PCA could not clearly differentiate between pure and adulterated samples (Figure 3A). On the other hand, PCA using five PCs subjected to metabolites data from beef-pork series cloud clearly differentiate authentic BMr, BMr adulterated PMr, and pure PMr with R^2^ = 0.680 and Q^2^ = 0.403 as depicted in Figure 3B. The tight cluster of the quality control (QC) samples in the PCA score plot confirmed the stability and the reproducibility of the LC-HRMS method in both sample series. PCA is an unsupervised pattern recognition that has been widely used in many fields of research including metabolomics to find out the similarity and dissimilarity among samples by reducing the number of original variables into few PCs. Samples with score plots appeared close to each other indicates high similarity and vice versa [19]. In this study, the obtained PCA showed that the higher the presence of adulterant in BMr, the closer the score plot to the score plot of adulterant both in beef-dog and beef-pork model. However, the separation among levels in adulterated samples is not clear enough such as a few of score plots are still overlapping. It might be caused by losing some information or data distortion when reducing the data dimensionality [16].

**Figure 4:**
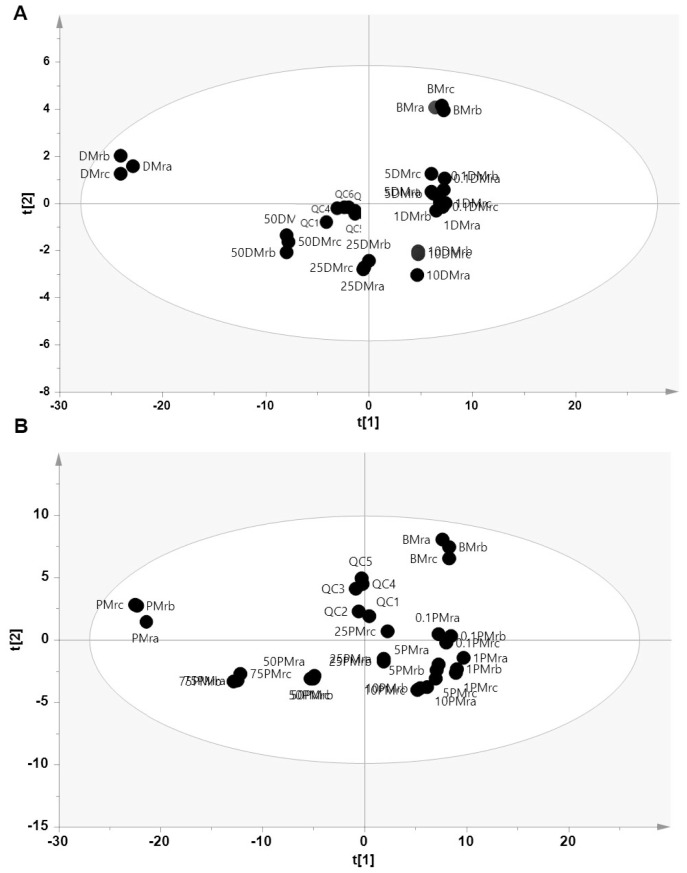
The score plot of partial least square-discriminant analysis to discriminate authentic beef meat from adulteration with dog meat (A) and pork meat (B). BMr = 100% beef; DMr = 100% dog; PMr = 100% pork, the number before samples code indicates the percentage of adulterant added in beef; QC = quality control samples.

To confirm the PCA results, supervised pattern recognition of PLS-DA was further used to discriminate and classify samples. PLS-DA using four latent variables (LV) clearly discriminated BMr, BMr adulterated DMr, and DMr samples with R^2^X = 0.797, R^2^Y = 0.961, and Q^2^ = 0.803. The score plot of PLS-DA in Figure 4A shows that the lowest level of DMr (0.1%) was clearly discriminated from the authentic BMr indicating a good performance of the PLS-DA model for discriminating adulterated samples at low concentration of adulterant. In addition, PLS-DA was also successfully used to discriminate between authentic and adulterated BMr with PMr, with high accuracy as depicted in Figure 4B. Using 9 LV, PLS-DA could detect and discriminate all adulterated samples from the authentic BMr samples with R^2^X = 0.774, R^2^Y = 0.960, and R^2^Y = 0.563. According to the PLS-DA score plot, the higher the level of the adulterants given, the closer the score plot to the adulterant meat, observed in both models. The table of misclassification test showed there is no misclassification found in both PLS-DA model of beef-dog and beef-pork series indicating the high accuracy of the classification models. In addition, the stability and reproducibility of the LC-HRMS method was confirmed by the tight cluster of QC samples.

Supervised PLS-DA is a combination of PLS regression and linear discriminant analysis. The PLS model was first built using the X matrix (independent variables), followed by Y matrix (dependent variables). Subsequently, the LV, also called factors were searched and used for classification. These LV made PLS-DA capable of returning better sample differentiation than using PCA. This result was in agreement with previous studies reporting the suitability of PLS-DA for sample classification of meat from different origins, for detection of pork in halal meats, and for metabolite differentiation of chicken during storage [14,28].

However, supervised pattern recognition such as PLS-DA is susceptible for model overfitting thereby could provide bias result. Therefore, such validation tests including permutation test and ROC test are often required to demonstrate the model validity. The permutation test performed using 999 permutations confirmed the validity of PLS-DA model both in BMr adulterated DMr and BMr adulterated PMr series. In the BMr adulterated DMr model, permutation using six components showed that the original variables had the highest value among all the permutated models with the intersection of Q^2^ value was between zero and below zero, (0.0, −0.231). Meanwhile, in the BMr adulterated PMr, permutation test using 8 components demonstrated the intersection value of Q^2^ of (0.0, −0.616) indicating model validity. The result of ROC test demonstrated the PLS-DA model validity supporting the permutation test. All samples were accurately classified in accordance with the result of misclassification test demonstrated by its AUC (area under the curve) of each class. The AUC value for each sample class obtained from both PLS-DA models was 1, exhibiting a good classification without misclassification [29].

### Identification of discriminating metabolites

The identification of variables having important role in samples differentiation which associated to discriminating metabolites samples differentiation was performed using the VIP value. These discriminating metabolites were important as potential biomarker candidates to discriminate non-halal meats in beef. The variables having an important role in samples differentiation are shown by the VIP value more than 1.0. The discriminating metabolites from VIP analysis of beef-dog series are shown in Supplementary Table S1. Among those metabolites, 19 metabolites were found to increase with the increase of DMr levels in BMr (Table 1). Meanwhile, the discriminating metabolites from beef-pork series are listed in Supplementary Table S2. Further analysis showed that 28 metabolites were observed to increase with the increase of pork level in beef (Table 2). The discriminating metabolites were cross confirmed using the ANOVA analysis [24]. Metabolites with p<0.05 were significant metabolites, that increase with increasing dog meat and pork levels in beef, respectively. The discriminating metabolites that increased in line with the level of adulterants consisted of various metabolites classes such as amino acids, organic acids, fatty acids, and other lipids. These significant metabolites could be used as the keys to detect and monitor the presence of dog meat and pork, respectively.

On the other hand, the area of metabolites acetyl-L-carnitine, DL-carnitine, C12-carnitine, and C14-carnitine were found to be high in BMr and increased as the level of BMr increased in both series. Carnitine is derived from amino acids, and it is endogenously synthesized in the liver, kidney, and brain from the amino acids of methionine and lysine. Carnitine is reported to be high in beef and it plays an important role in turning fat into energy. In addition, it eliminates some toxic compounds by transporting them out of mitochondria [30]. From the above results, it can be summarized that the utilization of VIP analysis is very beneficial to analyze metabolomics data from untargeted LC-HRMS to obtain discriminating metabolites to discriminate beef from dog and pork meat adulteration.

### Chemometrics of multivariate calibration

**Figure 5:**
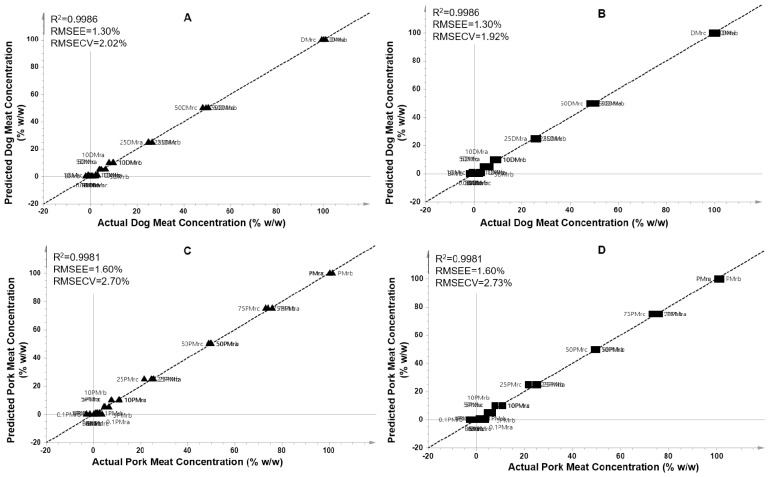
Plot of partial least square regression from beef-dog (A) and beef-pork dataset (C) and plot of orthogonal partial least square regression from beef-dog (B) and beef-pork (D) dataset.

The metabolites obtained from VIP analysis (VIP>1.0) were subjected to multivariate regression analysis using PLS regression and OPLS regression. Partial least square was successfully used to accurately predict the levels of DMr and PMr in the adulterated beef (% w/w), respectively. A high correlation between actual values and predicted values of DMr in adulterated BMr was obtained (Figure 5A and 5C). The PLS model had a high R^2^ value (0.9986) which is associated to its high model accuracy. In addition, the model had a low value of RMSEE (1.30%) and RMSECV (2.02%), associating to low error and high precision of the PLS model. In addition, PLS also successfully predict the levels of PMr present in BMr with high accuracy and high precision. The model provided R^2^ = 0.9981 with RMSEE of 1.60% and RMSECV of 2.70%. On the other hand, the regression of orthogonal PLS (OPLS) has also been widely applied for predicting target of analytes employing the orthogonal components. In this study, OPLS model was successfully detect and predict the adulterants of DMr and PMr in BMr as exemplified in Figure 5B and 5D. The obtained R^2^ was 0.9986 and 0.9981 with RMSEE of 1.30% and 1.60% for beef-dog and beef-pork series, respectively. In addition, the RMSECV value was 1.92% and 2.73% for beef-dog meat and beef-pork series, respectively.

Analysis on the residual plot both in PLS and OPLS models showed that all the residuals value were randomly spread and located at the standard deviation values between −4 and +4. This result indicated that the residuals were normally distributed, and no outliers were detected. The results showed that both PLS and OPLS regression had a similar performance in predicting targets. The results of PLS and OPLS demonstrated that the discriminating metabolites obtained from VIP analysis were good predictors to build a prediction model to determine levels of dog and pork meat present as adulterants in beef meat. Therefore, PLS and OPLS regression could be used to support the results of VIP analysis in investigating the discriminating metabolites potential as biomarker candidates to differentiate non-halal meats adulteration in halal meats.

PLS-based regression become the most multivariate calibration techniques applied in various analysis to predict the concentration of targeted analytes in the presence of other substances or matrices, because it could be used for accurate prediction of the target. The use of LV to correlate X-matrix and Y-matrix in the regression analysis results in high predicting capacity. Some studies have successfully applied PLS for halal authentication by detection of lard in edible fats and detection of pork in meat and meat-based products using spectroscopic techniques [31]. The discriminating metabolites of pork have been successfully confirmed as good predictors for pork adulteration by PLS and OPLS [25]. Another research also successfully used PLS to verify the role of discriminating metabolites in tea with different storage times. Result reveled that the discriminating metabolites were good predictors of storage time [32]. In our study, the result showed that both PLS and OPLS was potential for accurate prediction of DMr and PMr as the adulterants in halal beef meat.

In metabolomics study, it should be noted that the complexity of metabolomics data and its interpretation is not an easy task. Several factors such as the difference in diets, origins, environmental factors, meat preparation, and storage could affect the metabolites. In this study, we found a panel of metabolites which strongly associated with the increasing levels of dog meat and pork adulteration in minced beef. However, further investigation on the effect of factors that might affect the metabolites is required for future research to verify the metabolites found.

## CONCLUSION

The untargeted metabolomics approach using LC-HRMS combined with chemometrics could be an effective and powerful analytical technique for authentication of halal meats from non-halal meats adulteration. Further research using larger samples with various types of meats from different sources should be developed to warrant its reproducibility. Next, method standardization is required to obtain standard analytical method for halal testing of meats and meat-based food products.

## Figures and Tables

**Table 1:** Metabolites that increased in peak area with percentage of dog meat adulteration in beef obtained from variable importance for projections (VIP) value

No.	Compounds	Formula	RT (min)	Calculated m/z	Mass error	VIP value	p-value (ANOVA)
1	Hypoxanthine	C_5_H_4_N_4_O	0.918	136.03816	−2.61	1.99	4.69×10^−8^
2	5-Aminoimidazole ribotide	C_8_H_14_N_3_O_7_P	0.671	295.05803	3.72	1.92	6.26×10^−7^
3	2-[(5Z)-5-Tetradecenyl]cyclobutanone	C_18_H_32_O	20,336	264.24468	−2.39	1.87	4.16×10^−9^
4	L-Tyrosine	C_9_H_11_NO_3_	1.055	181.07339	−2.77	1.81	3.17×10^−7^
5	Ethyl myristate	C_16_H_32_O_2_	20.139	256.23973	−1.94	1.73	9.12×10^−5^
6	D-Pantothenic acid	C_9_H_17_NO_5_	2.808	219.11042	−1.17	1.68	0.0011
7	His-asp (L-histidyl-L-aspartic acid)	C_10_H_14_N_4_O_5_	0.691	270.09510	−4.89	1.66	0.0013
8	3-(Icosanoyloxy)-4-(trimethylammonio)butanoate	C_27_H_53_NO_4_	18.558	455.39655	−2.00	1.65	2.29×10^−4^
9	L-Gamma-glutamyl-L-leucine	C_11_H_20_N_2_O_5_	2.557	260.13707	−0.58	1.64	0.0076
10	Citric acid	C_6_H_8_O_7_	0.963	192.02630	−3.67	1.62	0.0064
11	2,3-Dihydroxybutanamide	C_4_H_9_NO_3_	0.775	119.05824	0.00	1.61	3.23×10^−5^
12	DL-Arginine	C_6_H_14_N_4_O_2_	0.704	174.11137	−1.78	1.58	0.0106
13	C8-Carnitine	C_15_H_29_NO_4_	10.400	287.20945	−0.72	1.34	3.59×10^−10^
14	1-Oleoyl-2-hydroxy-sn-glycero-3-PE	C_23_H_46_NO_7_P	18.863	479.30047	−1.50	1.34	7.18×10^−12^
15	9,12-Hexadecadienoylcarnitine	C_23_H_41_NO_4_	15.747	395.30301	−1.39	1.27	>0.05
16	L-Alpha-glycerylphosphorylcholine	C_8_H_20_NO_6_P	0.767	257.10231	−2.01	1.26	>0.05
17	L-(−)-Methionine	C_5_H_11_NO_2_S	0.867	149.05078	−1.81	1.24	>0.05
18	(2R)-1-{[(2-Aminoethoxy)(hydroxy)phosphoryl]oxy}-3-hydroxy-2-propanyl (11Z)-11-icosenoate	C_25_H_5_0NO_7_P	18.212	507.33151	−1.93	1.23	0.0118
19	Trans-3-indoleacrylic acid	C_11_H_9_NO_2_	3.485	187.06302	−1.66	1.21	>0.05

**Table 2:** Metabolites that increased in peak area with percentage of pork adulteration in beef meat obtained from variable importance for projections (VIP) value

No.	Compounds	Formula	RT	Calculated m/z	Mass error	VIP value	p-value (ANOVA)
1	Adrenic acid	C_22_H_36_O_2_	20.515	332.27050	−3.11	2.24	5.83×10^−8^
2	Nicotinamide	C_6_H_6_N_2_O	0.931	122.04789	−1.01	2.23	4.35×10^−10^
3	Linoleic acid	C_18_H_32_O_2_	19.556	280.23926	−3.45	2.22	2.00×10^−15^
4	Gluconic acid	C_6_H_12_O_7_	0.756	196.05750	−4.09	2.13	5.26×10^−5^
5	Arabinosylhypoxanthine	C_10_H_12_N_4_O_5_	1.311	268.08048	−1.08	2.10	7.42×10^−5^
6	(2R)-3-Hydroxy-2-[(9Z,12E)-9,12-octadecadienoyloxy]propyl 2-(trimethylammonio)ethyl phosphate	C_26_H_50_NO_7_P	18.123	519.33129	−2.32	2.00	2.28×10^−7^
7	C8-Carnitine	C_15_H_29_NO_4_	10.262	287.20950	−0.56	1.89	1.88×10^−10^
8	Acetyl-β-methylcholine	C_8_H_17_NO_2_	0.715	159.12582	−0.71	1.85	3.30×10^−5^
9	Dihydrothymine	C_5_H_8_N_2_O_2_	0.752	128.05863	0.38	1.83	0.006
10	1-Methylhistidine	C_7_H_11_N_3_O_2_	0.621	169.08496	−0.97	1.82	2.05×10^−5^
11	3beta-hydroxy-4beta-methyl-5alpha-cholest-7-ene-4alpha-carboxylic acid	C_29_H_48_O_3_	23.613	444.35927	−2.42	1.79	1.33×10^−14^
12	3-Hydroxy-3-[(3-methylbutanoyl)oxy]-4-(trimethylammonio)butanoate	C_12_H_23_NO_5_	1.286	261.15737	−0.97	1.75	7.57×10^−23^
13	1-Palmitoyl-2-hydroxy-sn-glycero-3-PE	C_21_H_44_NO_7_P	18.724	453.28470	−1.84	1.73	1.25×10^−4^
14	Oleic acid	C_18_H_34_O_2_	20.442	282.25542	−1.65	1.69	0.0029
15	Azelaic acid	C_9_H_16_O_4_	9.798	188.10413	−3.88	1.67	2.85×10^−4^
16	C14-Carnitine	C_21_H_41_NO_4_	15.874	371.30286	−1.89	1.67	>0.05
17	α-Aspartylphenylalanine	C_13_H_16_N_2_O_5_	3.714	280.10588	−0.16	1.63	>0.05
18	3-(Icosanoyloxy)-4-(trimethylammonio)butanoate	C_27_H_53_NO_4_	18.411	455.39654	−2.01	1.56	>0.05
19	4-Hydroxy-3-(sulfooxy)benzoic acid	C_7_H_6_O_7_S	0.184	233.98244	−4.18	1.50	0.0156
20	Dibenzylamine	C_14_H_15_N	7.171	197.12018	−1.35	1.48	0.0044
21	Arachidonic acid	C_20_H_32_O_2_	19.460	304.23990	−1.09	1.47	4.73×10^−5^
22	8Z,11Z,14Z-Eicosatrienoic acid	C_20_H_34_O_2_	20.004	306.25524	−2.09	1.41	>0.05
23	3-Hydroxyoctanoylcarnitine	C_15_H_29_NO_5_	7.910	303.20425	−1.07	1.35	0.0043
24	Choline	C_5_H_13_NO	19.642	103.09975	0.39	1.28	0.0032
25	(4S)-4-{[(9Z)-3-Hydroxy-9-hexadecenoyl]oxy}-4-(trimethylammonio)butanoate	C_23_H_43_NO_5_	15.420	413.31357	−1.35	1.25	>0.05
26	Stearamide	C_18_H_37_NO	20.165	283.28672	−2.81	1.20	7.20×10^−4^
27	Leu-Leu (N-L-Leucyl-L-leucine)	C_12_H_24_N_2_O_3_	5.827	244.17861	−0.32	1.15	>0.05
28	D-(+)-Proline	C_5_H_9_NO_2_	0.776	115.06331	−0.18	1.06	9.09×10^−4^

VIP, variable importance for projections; RT, retention time; ANOVA, analysis of variance.

## SUPPLEMENTARY MATERIAL

**Supplementary Table S1.** The metabolites with variable importance projections (VIP) value more than 1.0 to discriminate beef adulterated dog meat

**Supplementary Table S2.** The metabolites with variable importance projections (VIP) value more than 1.0 to discriminate beef adulterated pork meat

## References

[1] Martuscelli M, Serio A, Capezio O, Mastrocola D (2020). Safety, quality and analytical authentication of halal meat products, with particular emphasis on salami: a review. Foods.

[2] Alzeer J, Rieder U, Hadeed KA (2020). Good agricultural practices and its compatibility with Halal standards. Trends Food Sci Technol.

[3] Yuswan MH, Aizat WM, Desa MNM (2019). Improved gel-enhanced liquid chromatography-mass spectrometry by chemometrics for halal proteomics. Chemometr Intell Lab Syst.

[4] Hossain MAM, Uddin SMK, Sultana S (2022). Authentication of halal and kosher meat and meat products: analytical approaches, current progresses and future prospects. Crit Rev Food Sci Nutr.

[5] Pranata AW, Yuliana ND, Amalia L, Darmawan N (2021). Volatilomics for halal and non-halal meatball authentication using solid-phase microextraction–gas chromatography–mass spectrometry. Arab J Chem.

[6] Cao M, Han Q, Zhang J (2020). An untargeted and pseudotargeted metabolomic combination approach to identify differential markers to distinguish live from dead pork meat by liquid chromatography–mass spectrometry. J Chromatogr A.

[7] Rahayu WS, Rohman A, Martono S, Sudjadi S (2018). Application of FTIR spectroscopy and chemometrics for halal authentication of beef meatball adulterated with dog meat. Indones J Chem.

[8] Jiang H, Ru Y, Chen Q, Wang J, Xu L (2021). Near-infrared hyperspectral imaging for detection and visualization of offal adulteration in ground pork. Spectrochim Acta A Mol Biomol Spectrosc.

[9] Ghazali HH, Tukiran NA (2021). Analysis of pork adulteration in recycled frying oils using Raman spectroscopy. Malays J Halal Res.

[10] Orbayinah S, Hermawan A, Sismindari, Rohman A (2020). Detection of pork in meatballs using probe taqman real-time polymerase chain reaction. Food Res.

[11] Perestam AT, Fujisaki KK, Nava O, Hellberg RS (2017). Comparison of real-time PCR and ELISA-based methods for the detection of beef and pork in processed meat products. Food Control.

[12] Zhang M, Li Y, Zhang Y (2022). Rapid LC-MS/MS method for the detection of seven animal species in meat products. Food Chem.

[13] Muguruma Y, Nunome M, Inoue K (2022). A review on the foodomics based on liquid chromatography mass spectrometry. Chem Pharm Bull.

[14] Wang J, Xu Z, Zhang H (2021). Meat differentiation between pasture-fed and concentrate-fed sheep/goats by liquid chromatography quadrupole time-of-flight mass spectrometry combined with metabolomic and lipidomic profiling. Meat Sci.

[15] Belhaj MR, Lawler NG, Hoffman NJ (2021). Metabolomics and lipidomics: expanding the molecular landscape of exercise biology. Metabolites.

[16] Böhme K, Calo-Mata P, Barros-Velázquez J, Ortea I (2019). Recent applications of omics-based technologies to main topics in food authentication. Trends Analyt Chem.

[17] Selamat J, Rozani NAA, Murugesu S (2021). Application of the metabolomics approach in food authentication. Molecules.

[18] Cao S, Du H, Tang B, Xi C, Chen Z (2021). Non-target metabolomics based on high-resolution mass spectrometry combined with chemometric analysis for discriminating geographical origins of Rhizoma Coptidis. Microchem J.

[19] Paul A, Harrington PDB (2021). Chemometric applications in metabolomic studies using chromatography-mass spectrometry. Trends Analyt Chem.

[20] Balkir P, Kemahlioglu K, Yucel U (2021). Foodomics: a new approach in food quality and safety. Trends Food Sci Technol.

[21] Panseri S, Arioli F, Pavlovic R (2022). Impact of irradiation on metabolomics profile of ground meat and its implications toward food safety. LWT.

[22] Abbas N, Ali A, Kumari S (2020). Untargeted-metabolomics differentiation between poultry samples slaughtered with and without detaching spinal cord. Arab J Chem.

[23] Sidwick KL, Johnson AE, Adam CD, Pereira L, Thompson DF (2017). Use of liquid chromatography quadrupole time-of-flight mass spectrometry and metabonomic profiling to differentiate between normally slaughtered and dead on arrival poultry meat. Anal Chem.

[24] Trivedi DK, Hollywood KA, Rattray NJW (2016). Meat, the metabolites: an integrated metabolite profiling and lipidomics approach for the detection of the adulteration of beef with pork. Analyst.

[25] Windarsih A, Riswanto FDO, Bakar NKA, Yuliana ND, Dachriyanus, Rohman A (2022). Detection of pork in beef meatballs using LC-HRMS based untargeted metabolomics and chemometrics for halal authentication. Molecules.

[26] Kausar T, Hanan E, Ayob O, Praween B, Azad Z (2019). A review on functional ingredients in red meat products. Bioinformation.

[27] Elbir Z, Oz F (2021). The assessment of commercial beef and chicken bouillons in terms of heterocyclic aromatic amines and some of their precursors. Int J Food Sci Technol.

[28] Leng T, Li F, Xiong L, Xiong Q, Zhu M, Chen Y (2020). Quantitative detection of binary and ternary adulteration of minced beef meat with pork and duck meat by NIR combined with chemometrics. Food Control.

[29] Guntarti A, Ayu Purbowati Z (2019). Analysis of dog fat in beef sausage using FTIR (Fourier Transform Infrared) combined with chemometrics. Pharmaciana.

[30] Rospond B, Chlopicka J (2013). The biological function of L-carnitine and its content in the particular food examples. Przegl Lek.

[31] Pebriana RB, Rohman A, Lukitaningsih E, Sudjadi (2017). Development of FTIR spectroscopy in combination with chemometrics for analysis of rat meat in beef sausage employing three lipid extraction systems. Int J Food Prop.

[32] Shen S, Huang J, Li T (2022). Untargeted and targeted metabolomics reveals potential marker compounds of an tea during storage. LWT.

