# Supplementary material for: Untargeted metabolomics using liquid chromatography-high resolution mass spectrometry and chemometrics for analysis of non-halal meats adulteration in beef meat
Source: Anim Biosci. 2024 Jan 14;37(5):918–28. doi: 10.5713/ab.23.0238 (PMC11065716; doi:10.5713/ab.23.0238)
Supplement: Supplementary file 1 [file ab-23-0238-Supplementary-Table-1.pdf]

Table S1. The metabolites with variable importance projections (VIP) value more than 1.0 to discriminate beef adulterated dog meat

No.	Compounds	VIP value	Formula	Mass error (ppm)	Calculated m/z	Retention time (ppm)
1	Hypoxanthine	2.05	C5H4N4O	-2.61	136.03816	0.918
2	5-aminoimidazole ribotide	1.94	C8H14N3O7P	3.72	295.05803	0.671
3	L-Tyrosine	1.92	C9H11NO3	-2.77	181.07339	1.055
4	2-[(5Z)-5-tetradecenyl]cyclobutanone	1.92	C18H32O	-2.39	264.24468	20.336
5	cis-5-Tetradecenoylcarnitine	1.86	C21H39NO4	-1.91	369.28720	15.373
6	Ethyl myristate	1.76	C16H32O2	-1.94	256.23973	20.139
7	D-PANTOTHENIC ACID	1.75	C9H17NO5	-1.17	219.11042	2.808
8	his-asp	1.73	C10H14N4O5	-4.89	270.09510	0.691
9	C14-Carnitine	1.71	C21H41NO4	-1.98	371.30282	16.052
10	D-lysopine	1.70	C9H18N2O4	-1.19	218.12640	2.075
11	Citric acid	1.68	C6H8O7	-3.67	192.02630	0.963
12	L-gamma-Glutamyl-L-leucine	1.67	C11H20N2O5	-0.58	260.13707	2.557
13	2,3-Dihydroxybutanamide	1.65	C4H9NO3	0.00	119.05824	0.775
14	3-(Icosanoyloxy)-4-(trimethylammonio)butanoate	1.64	C27H53NO4	-2.00	455.39655	18.558
15	Piperidine	1.63	C5H11N	2.43	85.08936	1.181
16	DL-Arginine	1.60	C6H14N4O2	-1.78	174.11137	0.704
17	D-(+)-Galactose	1.59	C6H12O6	-2.24	180.06299	0.686
18	1-Linoleoyl glycerol	1.55	C21H38O4	-3.29	354.27584	18.838
19	Isoleucine	1.53	C6H13NO2	-1.97	131.09437	1.189
20	Acetyl-β-methylcholine	1.51	C8H17NO2	-096	159.12578	0.704
21	D-Gluconic acid	1.50	C6H12O7	-3.37	196.05764	0.747
22	Acetyl-L-carnitine	1.49	C9H17NO4	-2.91	203.11517	0.711
23	2-methylbutyrylcarnitine	1.47	C12H23NO4	-0.91	245.16249	4.26
24	N-Stearoyltyrosine	1.46	C27H45NO4	-1.93	447.33400	16.729
25	D-(+)-Proline	1.42	C5H9NO2	-0.19	115.06331	0.755
26	3-hydroxytetradecanoylcarnitine	1.39	C21H41NO5	-1.74	387.29780	15.022
27	LysoPC(P-18:0)	1.38	C26H54NO6P	-2.49	507.36761	19.662
28	Oleic acid	1.37	C18H34O2	-2.57	282.25516	20.348
29	L-(-)-Methionine	1.37	C5H11NO2S	-1.81	149.05078	0.867
30	trans-3-Indoleacrylic acid	1.37	C11H9NO2	-1.66	187.06302	3.485
31	C8-Carnitine	1.36	C15H29NO4	-0.72	287.20945	10.4
32	1-Oleoyl-2-hydroxy-sn-glycero-3-PE	1.35	C23H46NO7P	-1.50	479.30047	18.863
33	1-[(11Z)-octadecenoyl]-sn-glycero-3-phosphocholine	1.35	C26H52NO7P	-2.26	521.34696	18.718
34	(2R)-1-{[(2-Aminoethoxy)(hydroxy)phosphoryl]oxy}-3-hydroxy-2-propanyl (4Z,7Z,10Z,13Z,16Z)-4,7,10,13,16-docosapentaenoate	1.32	C27H46NO7P	-1.59	527.30035	18.295
35	L-alpha-Glycerylphosphorylcholine	1.32	C8H20NO6P	-2.01	257.10231	0.767
36	Linoleic acid	1.30	C18H32O2	-3.43	280.23927	19.482
37	9,12-Hexadecadienoylcarnitine	1.30	C23H41NO4	-1.39	395.30301	15.747
38	α-Aspartylphenylalanine	1.27	C13H16N2O5	-0.40	280.10581	3.755

39	(2R)-1-{[(2-Aminoethoxy)(hydroxy)phosphoryl]oxy}-3-hydroxy-2-propanyl (11Z)-11-icosenoate	1.26	C25H50NO7P	-1.93	507.33151	18.212
40	Succinylcholine	1.26	C14H30N2O4	3.21	290.22149	19.687
41	α-Eleostearic acid	1.21	C18H30O2	-2.64	278.22385	18.886
42	D-ribose 5-phosphate	1.21	C5H11O8P	-1.44	230.01882	0.779
43	2-Amino-1,3,4-octadecanetriol	1.21	C18H39NO3	-1.61	317.29248	14.105
44	Benzoic acid	1.19	C7H6O2	2.93	122.03714	1.056
45	Tiglylcarnitine	1.18	C12H21NO4	-0.52	243.14693	3.864
46	(R)-3-hydroxybutyrylcarnitine	1.18	C11H21NO5	-1.97	247.14149	0.724
47	Stearic acid	1.16	C18H36O2	-0.20	284.27147	21.931
48	1-arachidonoyl-sn-glycero-3-phosphoethanolamine	1.16	C25H44NO7P	-1.04	501.28502	18.089
49	Palmitoleic acid	1.14	C16H30O2	-1.64	254.22416	19.105
50	2-Amino-3-hydroxy-15-methyl-1-hexadecanesulfonic acid	1.14	C17H37NO4S	3.09	351.24542	18.498
51	Prolylleucine	1.10	C11H20N2O3	-2.10	228.14691	0.732
52	3-Methylhistidine	1.10	C7H11N3O2	-1.70	169.08488	0.669
53	3,5,7-Octatriyn-1-ol	1.10	C8H6O	-0.68	118.04178	1.04
54	Betaine	1.08	C5H11NO2	-0.86	117.07888	0.724
55	3-hydroxyoctanoylcarnitine	1.04	C15H29NO5	-1.19	303.20421	8.048
56	Palmitoylcarnitine	1.04	C23H45NO4	-2.16	399.33399	17.083
57	3, 5-Tetradecadiencarnitine	1.03	C21H37NO4	-1.91	367.27156	14.599
58	3-Methylsulfolene	1.03	C5H8O2S	-1.82	132.02426	0.875
59	(2S)-2,3-Dihydroxypropyl (9Z)-9-hexadecenoate	1.02	C19H36O4	-1.19	328.26097	20.329
60	2-Acetyl-6-Methylpyridine	1.01	C8H9NO	-1.01	135.06828	1.042
