# Supplementary material for: Untargeted metabolomics using liquid chromatography-high resolution mass spectrometry and chemometrics for analysis of non-halal meats adulteration in beef meat
Source: Anim Biosci. 2024 Jan 14;37(5):918–28. doi: 10.5713/ab.23.0238 (PMC11065716; doi:10.5713/ab.23.0238)
Supplement: Supplementary file 2 [file ab-23-0238-Supplementary-Table-2.pdf]

Table S2. The metabolites with variable importance projections (VIP) value more than 1.0 to discriminate beef adulterated pork meat

No.	Compounds	VIP value	Formula	Mass error (ppm)	Calculated m/z	Retention time (ppm)
1	Acetyl-L-carnitine	2.34	C9H17NO4	-2.58	203.11523	0.723
2	Adrenic acid	2.24	C22H36O2	-3.11	332.27050	20.515
3	Nicotinamide	2.23	C6H6N2O	-1.01	122.04789	0.931
4	Linoleic acid	2.22	C18H32O2	-3.45	280.23926	19.556
5	DL-Carnitine	2.22	C7H15NO3	-2.16	161.10485	0.715
6	Gluconic acid	2.13	C6H12O7	-4.09	196.05750	0.756
7	Arabinosylhypoxanthine	2.10	C10H12N4O5	-1.08	268.08048	1.311
8	Betaine	2.10	C5H11NO2	-0.83	117.07888	0.744
9	C12-Carnitine	2.09	C19H37NO4	-1.55	343.27173	14.543
10	2,4-Diacetamido-2,4,6-trideoxy-alpha-D-mannopyranose	2.02	C10H18N2O5	-1.40	246.12123	2.534
11	(2R)-3-Hydroxy-2-[(9Z,12E)-9,12-octadecadienoyloxy]propyl 2-(trimethylammonio)ethyl phosphate	1.99	C26H50NO7P	-2.32	519.33129	18.123
12	C8-Carnitine	1.89	C15H29NO4	-0.56	287.20950	10.262
13	Hypoxanthine	1.89	C5H4N4O	-2.72	136.03814	0.922
14	Acetyl-β-methylcholine	1.85	C8H17NO2	-0.71	159.12582	0.715
15	Dihydrothymine	1.83	C5H8N2O2	0.38	128.05863	0.752
16	1-Methylhistidine	1.82	C7H11N3O2	-0.97	169.08496	0.621
17	Guanine	1.82	C5H5N5O	-1.66	151.04916	1.310
18	3beta-hydroxy-4beta-methyl-5alpha-cholest-7-ene-4alpha-carboxylic acid	1.79	C29H48O3	-2.42	444.35927	23.613
19	3-Hydroxy-3-[(3-methylbutanoyl)oxy]-4-(trimethylammonio)butanoate	1.75	C12H23NO5	-0.97	261.15737	1.286
20	1-Palmitoyl-2-hydroxy-sn-glycero-3-PE	1.73	C21H44NO7P	-1.84	453.28470	18.724
21	O-heptanoylcarnitine	1.69	C14H27NO4	-0.13	273.19397	8.120
22	Oleic acid	1.69	C18H34O2	-1.65	282.25542	20.442
23	Azelaic acid	1.67	C9H16O4	-3.88	188.10413	9.798
24	Decanoylcarnitine	1.67	C17H33NO4	-0.98	315.24065	12.774
25	C14-Carnitine	1.67	C21H41NO4	-1.89	371.30286	15.874
26	α-Aspartylphenylalanine	1.63	C13H16N2O5	-0.16	280.10588	3.714
27	L-Tyrosine	1.58	C9H11NO3	-4.91	181.07300	1.069
28	3-(Icosanoyloxy)-4-(trimethylammonio)butanoate	1.56	C27H53NO4	-2.01	455.39654	18.411
29	LysoPC(22:4(7Z,10Z,13Z,16Z))	1.51	C30H54NO7P	-2.46	571.36238	18.923
30	4-Hydroxy-3-(sulfooxy)benzoic acid	1.50	C7H6O7S	-4.18	233.98244	0.184
31	Dibenzylamine	1.48	C14H15N	-1.35	197.12018	7.171
32	Arachidonic acid	1.47	C20H32O2	-1.09	304.23990	19.460
33	Hexanoylcarnitine	1.43	C13H25NO4	-1.01	259.17810	6.366
34	Margaric acid	1.41	C17H34O2	-0.57	270.25573	21.058
35	8Z,11Z,14Z-Eicosatrienoic acid	1.41	C20H34O2	-2.09	306.25524	20.004
36	cis-5-Tetradecenoylcarnitine	1.40	C21H39NO4	-1.55	369.28734	15.197
37	2-Aminooctadec-4-yne-1,3-diol	1.37	C18H35NO2	-0.88	297.26652	15.532
38	3-hydroxyoctanoylcarnitine	1.35	C15H29NO5	-1.07	303.20425	7.910

39	4-(Hydroxymethyl)benzenesulfonic acid	1.32	C7H8O4S	-3.69	188.01364	5.732
40	Choline	1.28	C5H13NO	0.39	103.09975	19.642
41	3-Methylsulfolene	1.27	C5H8O2S	-2.04	132.02423	0.878
42	D-ribose 5-phosphate	1.27	C5H11O8P	-2.28	230.01863	0.792
43	N-(tert-Butoxycarbonyl)-L-leucine	1.27	C11H21NO4	-1.95	231.14661	2.103
44	1-(1Z-hexadecenyl)-2-arachidonoyl-sn-glycero-3-phosphoethanolamine	1.26	C41H74NO7P	-3.53	723.51774	21.150
45	(4S)-4-{[(9Z)-3-Hydroxy-9-hexadecenoyl]oxy}-4-(trimethylammonio)butanoate	1.25	C23H43NO5	-1.35	413.31357	15.420
46	Stearamide	1.20	C18H37NO	-2.81	283.28672	20.165
47	2-methyl-5-pyridin-2-yl-7-(trifluoromethyl)pyrazolo[1,5-a]pyrimidine	1.19	C13H9F3N4	-1.22	278.07759	0.674
48	(2R)-3-{[(2-Aminoethoxy)(hydroxy)phosphoryl]oxy}-2-hydroxypropyl (11Z,14Z,17Z)-11,14,17-icosatrienoate	1.16	C25H46NO7P	-1.52	503.30042	18.444
49	Leu-Leu	1.15	C12H24N2O3	-0.32	244.17861	5.827
50	Suberic acid	1.13	C8H14O4	-4.72	174.08839	7.987
51	Ethyl myristate	1.12	C16H32O2	-1.18	256.23993	20.235
52	γ-Linolenic acid	1.10	C18H30O2	-1.88	278.22406	18.942
53	Z-Leu-OH	1.09	C14H19NO4	-0.20	265.13135	4.752
54	9,12-Hexadecadienoylcarnitine	1.08	C23H41NO4	-1,41	395.30300	15.571
55	DL-Glutamine	1.06	C5H10N2O3	-1.10	146.06898	0.696
56	D-(+)-Proline	1.06	C5H9NO2	-0.18	115.06331	0.776
57	1-Oleoyl-2-hydroxy-sn-glycero-3-PE	1.05	C23H46NO7P	-0.70	479.30085	18.682
58	Leucylproline	1.04	C11H20N2O3	-0.12	228.14736	2.809
59	trans-p-Coumaraldehyde	1.03	C9H8O2	-1.32	148.05223	1.878
60	L-Phenylalanine	1.03	C9H11NO2	-1.17	165.07879	1.872
61	Creatine	1.02	C4H9N3O2	-2.26	131.06918	0.749
62	Valeric acid	1.02	C5H10O2	2.02	102.06829	8.176
